# Dental Societies

**Published:** 1890-05

**Authors:** 


### Editorial.

### DENTAL SOCIETIES.

The past few years have been eventful ones in the history of dentistry. No one thing has contributed more towards the development of this specialty into a science—and a recognized place among the medical specialties—than the excellent work done by the dental societies.

The society has, in the highest degree, been the birthplace and main-stay of dental literature. Had it not been for its fostering care, our literature never would have reached the high plane upon which it rests to-day. The emulation between the different societies has been the most powerful lever to draw out the busy practitioner and secure from him literary efforts which have tended to better the individual and elevate his fellows. Dentists as a class are not inclined to literary work. They labor at the chair all day, and when the evening comes they much prefer the intercourse of the family circle to the solitude of the study; and it is only the promise to prepare a paper for some association, which is made in most instances under great pressure, that spurs the physically weary man to exert himself mentally for the benefit of his brother specialists.

There is a small class of dentists, however, who write articles for publication from other motives, but they are not the ones who are best prepared to instruct, for, as a rule, they are not practical or successful men; and the very fact that they have the time to write, in many instances is proof of their unfitness to do so. In every community are to be found busy men who are most thoroughly prepared to furnish the very best literature, but who seldom or never write anything. Their time is too valuable to spend in putting thoughts on paper when they are coining their efforts into gold. If they are ever induced to present anything to the profession, it is through the earnest solicitations and sometimes importunities of the executive committee of some society. Theirs is seldom, if ever, a voluntary effort; and if estimated by the actual loss of time would come high in dollars and cents, so high, indeed, that no journal in this country could afford to pay for it; nevertheless, it is given without cost to some society and appears in its published proceedings. These papers represent the very cream of our dental literature, for they bristle all over with practical points that could not be obtained in any other way. Their value is always in the inverse ratio to their production. The society, therefore, deserves the fullest praise for its success in bringing out the very class of men who are best fitted by experience and ability to furnish our literature.

Not only does the society give us our leading papers, but it also furnishes an opportunity for the exchange of opinions under the head of "Discussions." It is there that the dentist is most at home, and the ideas that are drawn out under the inspiration of the favorable surroundings are most valuable contributions to our knowledge. It is true that drift-wood and repetition sometimes creep into discussions, but if well edited, they form the most valuable portion of our literature; little bits, practical points, and comparisons of methods are put in close space. The paper in this issue, on "Some Practical Points learned at Society Meetings," is proof of the truth of these statements.

POST-GRADUATE COURSES.

The society forms a post-graduate course of instruction for the busy practitioner, in which he may secure many advantages, and add to his knowledge of methods of practice. Too much stress cannot be laid upon the value of society meetings, and it has been with the full knowledge of their importance that this journal has steadfastly worked to secure the exclusive control of the proceedings of the leading dental societies of this country and Europe. How well it has succeeded may be known by the fact that the INTERNATIONAL stands to-day as the official organ of the Academy of Dental Science of Boston, the New York Odontological Society, the Odontological Society of Pennsylvania, the Chicago Dental Club, and the American Dental Society of Europe. In addition it contains the exclusive reports of the meetings of the New Jersey State Dental Society, the Massachusetts State Dental Society, the Union Meeting of the New England Dental Society, the Connecticut State Dental Association, and the Connecticut Valley Dental Society; the Pennsylvania State Dental Society, and the Maryland State Dental Society. It has also received reports through its numerous foreign correspondents from several foreign dental societies. To give some idea of the amount of matter presented at these meetings during the past year we will refer to Volume X. of the INTERNATIONAL DENTAL JOURNAL, 1889, which contains 766 pages of original matter and a topical index of 52 pages. By reference to the latter, it will be seen that during the year four thousand two hundred and thirty subjects were discussed by nearly two hundred dentists,—an average of over three pages to each person. The volume also contains eighty-one illustrations, including three photogravure plates, all the efforts of dentists in dental meetings, and, altogether, it forms a lasting record of the good work done in the leading dental societies in this country and Europe during the year.

In this review of our dental societies we do not desire to overlook the work of the several district societies of New York State, especially that of the First District Society of New York City and of the American Dental Association, reported in the *Cosmos*, nor of the Illinois State and the Chicago Dental Societies, the reports of which form the basis of matter for the *Dental Review*.

### DENTAL SECTION OF THE AMERICAN MEDICAL ASSOCIATION.

Then, again, within the last few years there has been a steady growth in the direction of the Dental Section of the American Medical Association. The meeting last year, at Newport, was a most successful one, and the meeting this year, to be held at Nashville in May, promises to be the most brilliant one yet. The programme, as published in the April number of this journal, contains a splendid array of talent. The report of this meeting belongs exclusively to the journal of the American Medical Association, which has a circulation of over five thousand. It is needless to say that the papers of the Dental Section hold their own in importance and scientific interest with those of the other sections. The paper by Dr. Andrews, in the journal of the Association of April 12, illustrated by a full-page plate showing the predisposing causes that lead to decay, and the other able papers read before this section, and which have appeared during the year, cannot help but have an influence upon the status of dentistry, and tend to interest medical men in dental science, and instruct them in a specialty in medicine, of which general practitioners have heretofore known but little.

No single movement that has been undertaken in late years by dentists has promised more towards bringing together what has heretofore been considered two separate professions than this section in the American Medical Association. Medical men are beginning to learn that dentistry is something more than a mere trade, and dentists are really finding out that dentistry has many things in common with medicine, while all are willing to admit that dentistry, practised as a specialty in medicine, is one of the nicest specialties in existence.

